# Supplementary material for: Large-scale characterisation of the pregnancy vaginal microbiome and sialidase activity in a low-risk Chinese population
Source: NPJ Biofilms Microbiomes. 2021 Dec 20;7:89. doi: 10.1038/s41522-021-00261-0 (PMC8688454; doi:10.1038/s41522-021-00261-0)
Supplement: Supplementary file 1 — Supplementary Information [file 41522_2021_261_MOESM1_ESM.pdf]

Supplementary Materials

Large-scale characterisation of the pregnancy vaginal microbiome and sialidase activity in a low-risk Chinese population

Sherrianne Ng[1,2+], Muxuan Chen[3,4+], Samit Kundu[1,2], Xuefei Wang[5], Zuyi Zhou[3,4], Zhongdaixi Zheng[6], Wei Qing[3,4], Huafang Sheng[3,4], Yan Wang[5], Yan He[3,4], Phillip R. Bennett[1,2], David A. MacIntyre[1,2*] and Hongwei Zhou[3,4,7*].

Contains:

Supplementary Figures 1-7

Supplementary Tables 1-5

Supplementary Figures

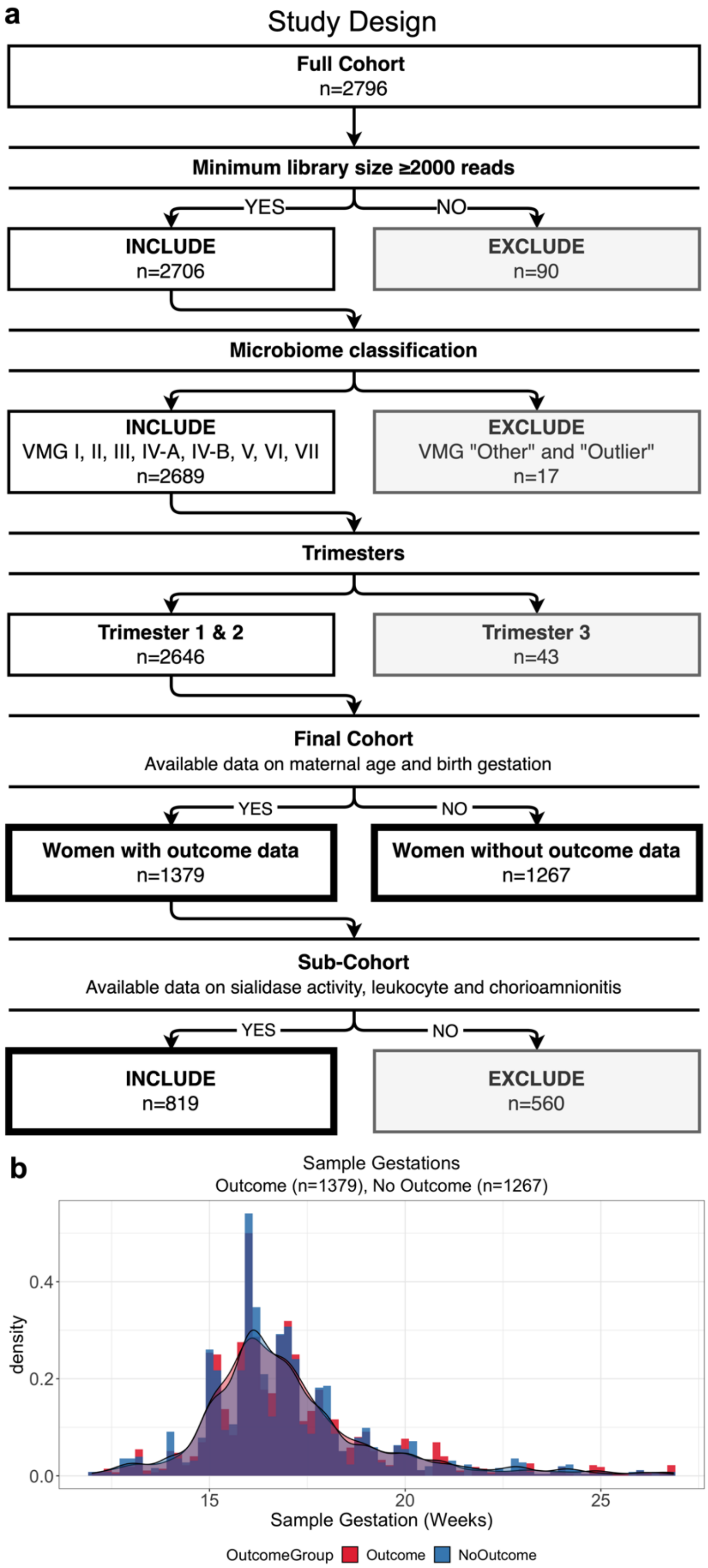

Supplementary Figure 1. (a) Study design of women included in final cohort analysis comparing women with and without outcome data and sub-cohort analysis of women with available maternal and neonatal data. (b) Density plot showing sample gestation of women with (red) and without (blue) outcome included in final cohort analysis.

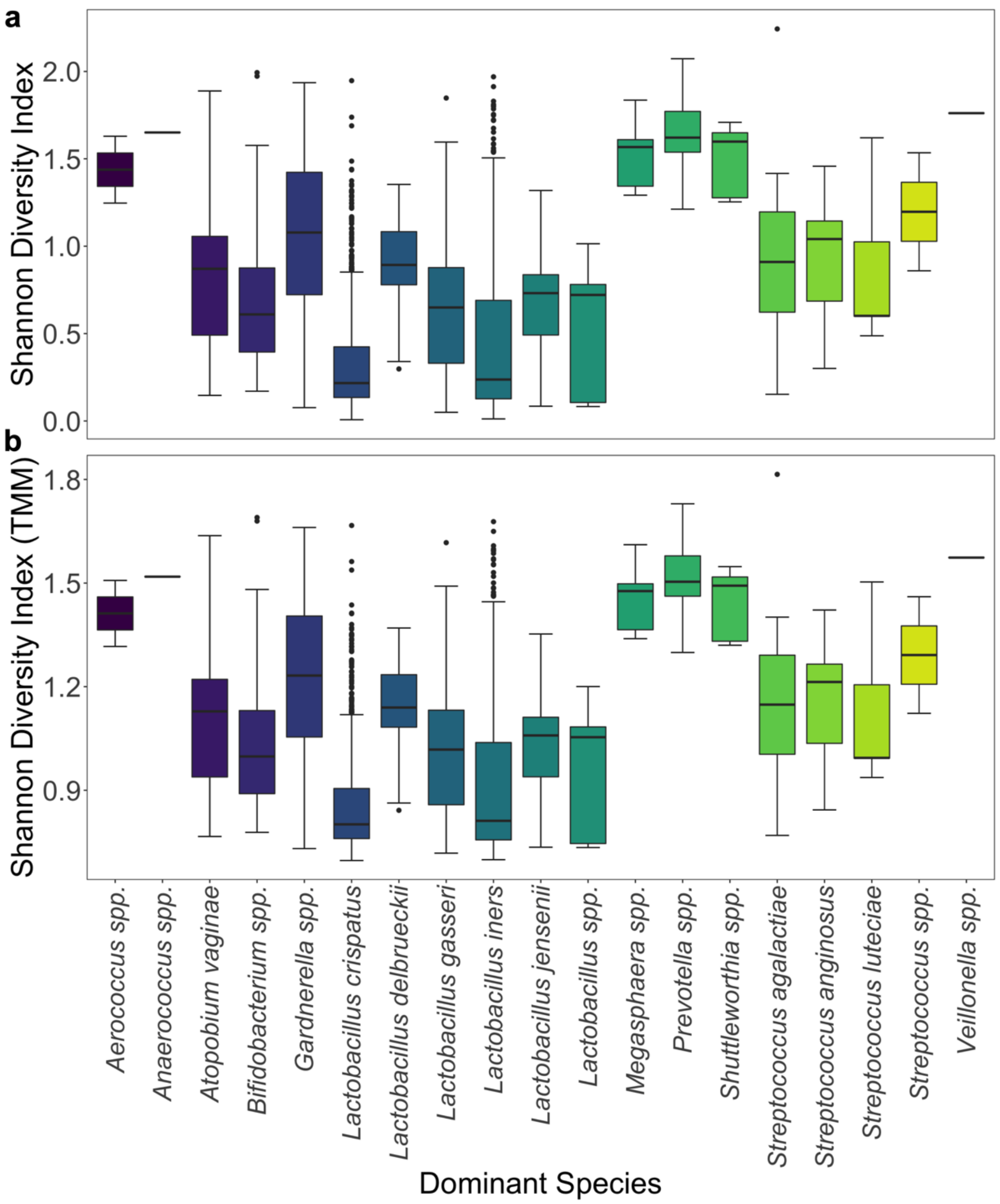

Supplementary Figure 2. Comparison of Shannon diversity index (a) before and (b) after trimmed mean of M-values (TMM) normalisation. The bounds of the box represent the first and third quartiles, centre line represents the median, and whiskers show min-to-max values.

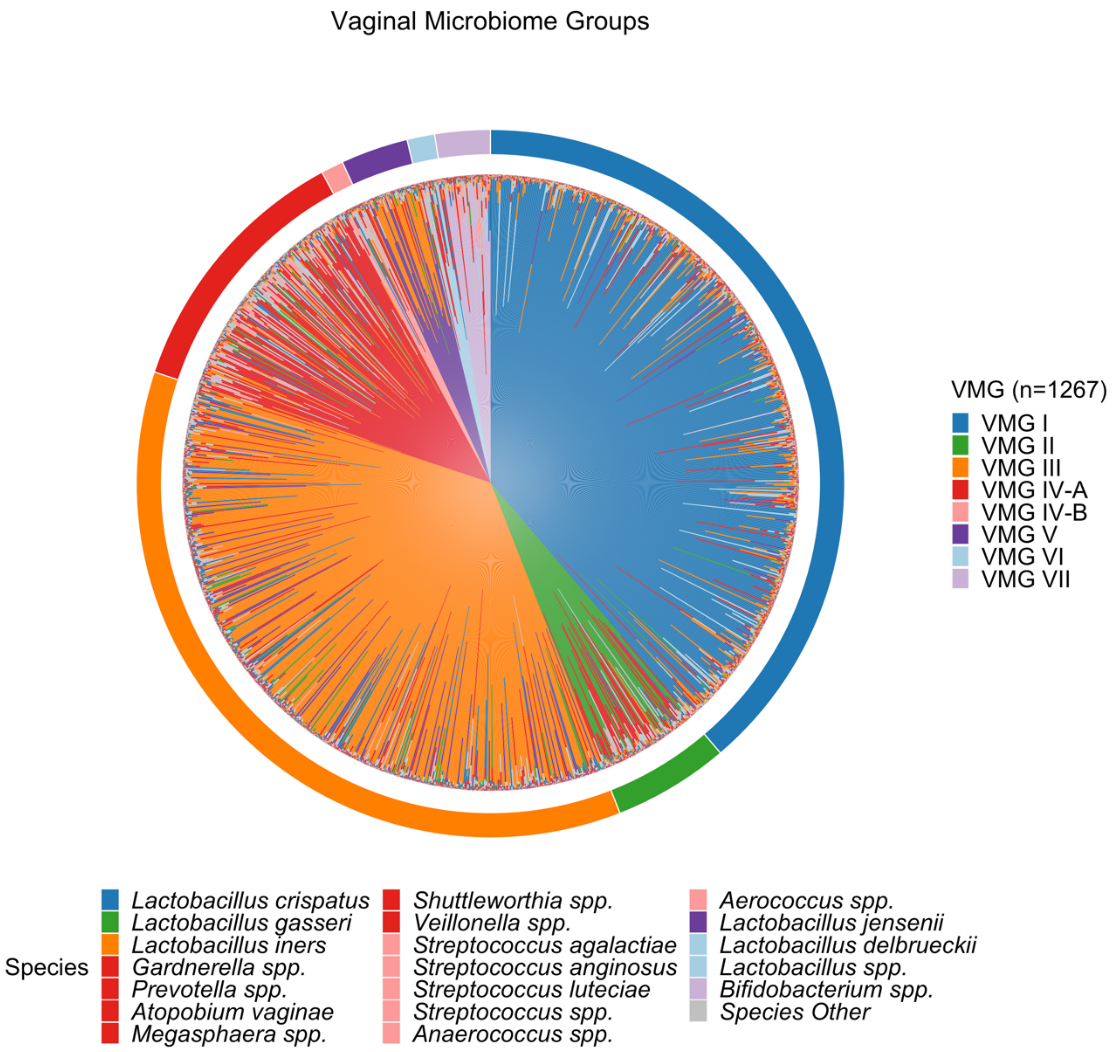

Supplementary Figure 3. Vaginal microbiome groups of women without outcome data. Radial plot showing vaginal microbiome groups (VMG) of women without outcome data. Species classified into VMG I (*L. crispatus* dominated, blue); II (*L. gasseri* dominated, green); III (*L. iners* dominated, orange); IV-A (BV-associated, red); IV-B (pathobionts, pink); V (*L. jensenii* dominated, purple); VI (Other *Lactobacillus*, light blue) and VII (*Bifodobacterium* dominated, light purple). Outcome data was defined as availability of maternal age and birth gestation.

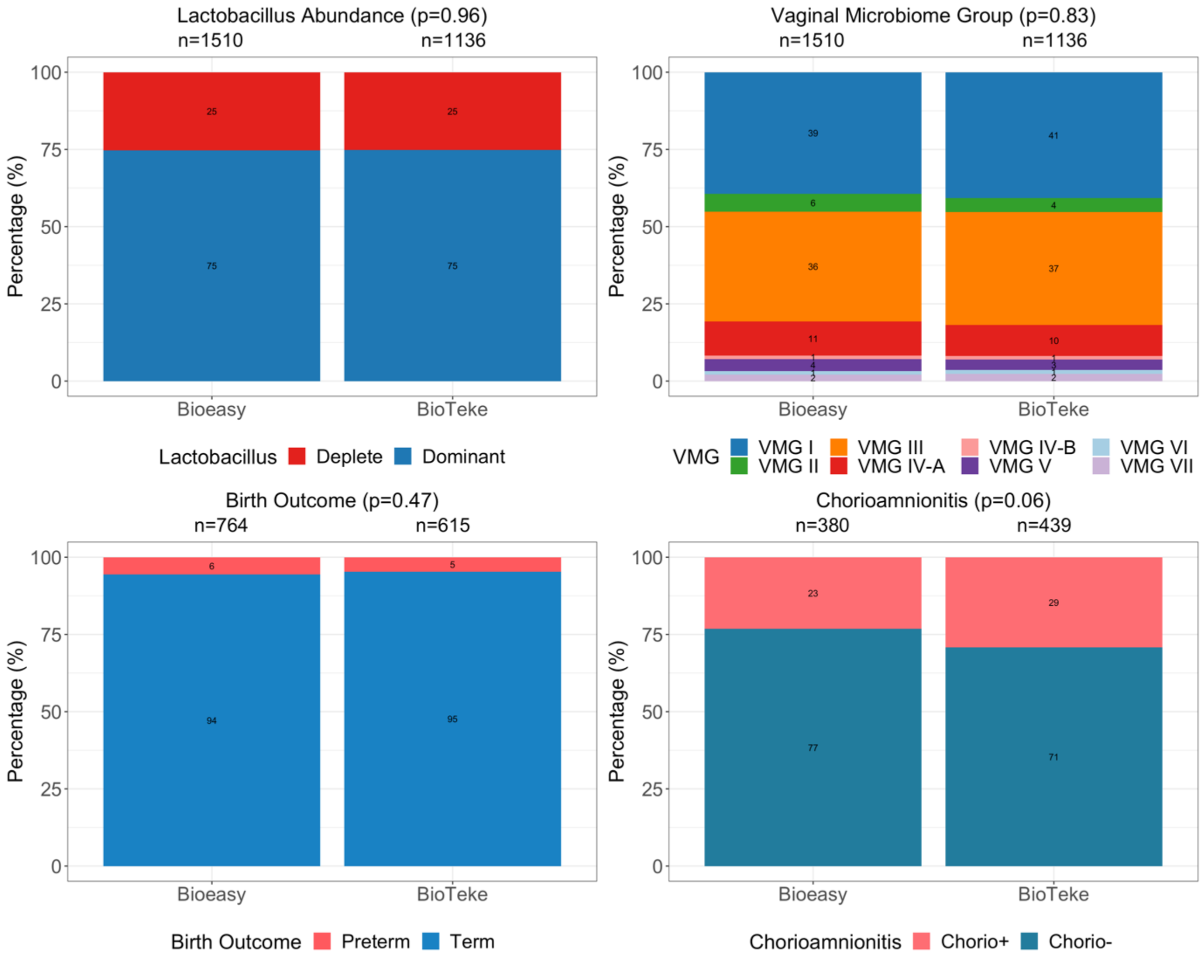

Supplementary Figure 4. Comparison of *Lactobacillus* abundance, VMG, birth outcome and chorioamnionitis proportions between samples extracted using manual (BioTeke) and automated (Bioeasy) DNA extraction kits. Statistical significance based on Fisher's Exact Test.

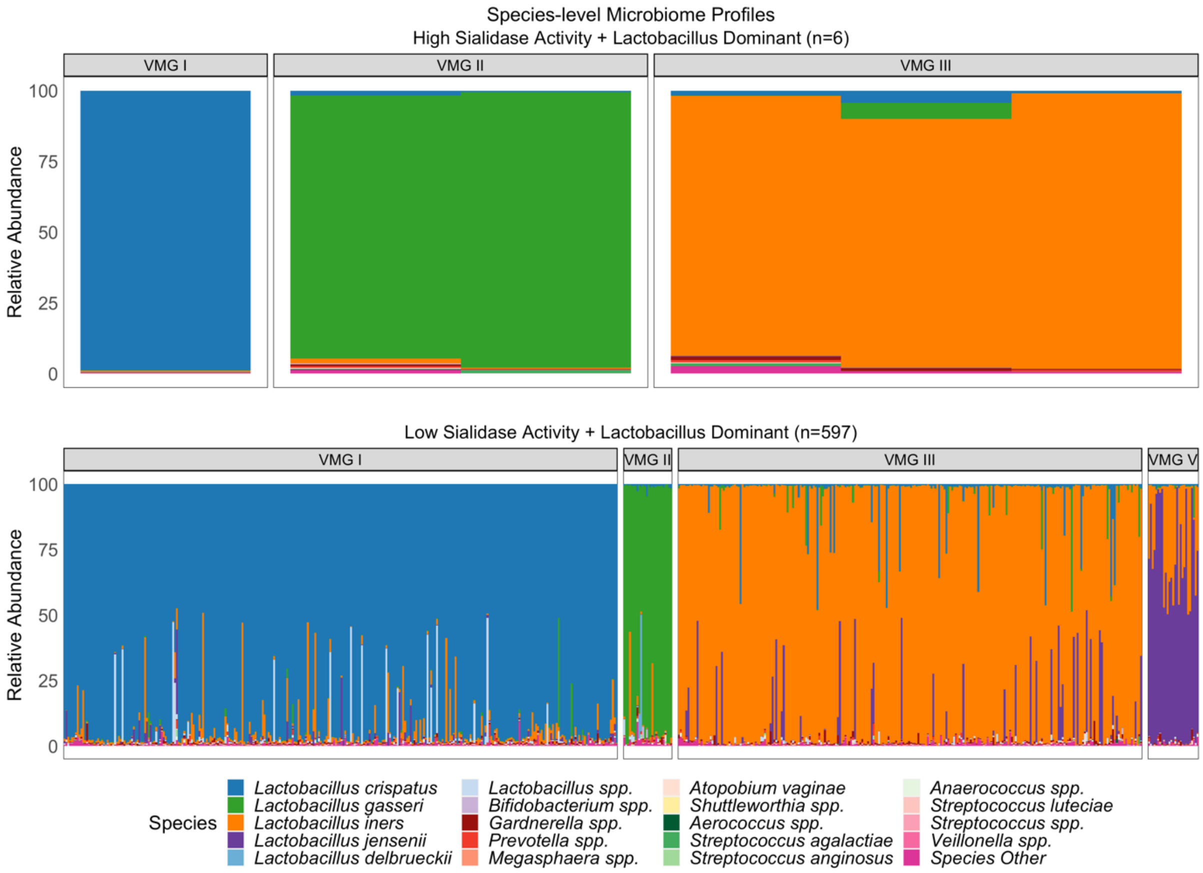

Supplementary Figure 5. *Lactobacillus* dominant microbiome profiles of women with high and low sialidase activity. The microbiome profiles of women with *Lactobacillus* dominance and high or low sialidase activity dominated by Vaginal Microbiome Group (VMG) I (*L. crispatus*, blue), II (*L. gasseri*, green), III (*L. iners*, orange) and/or V (*L. jensenii*, purple).

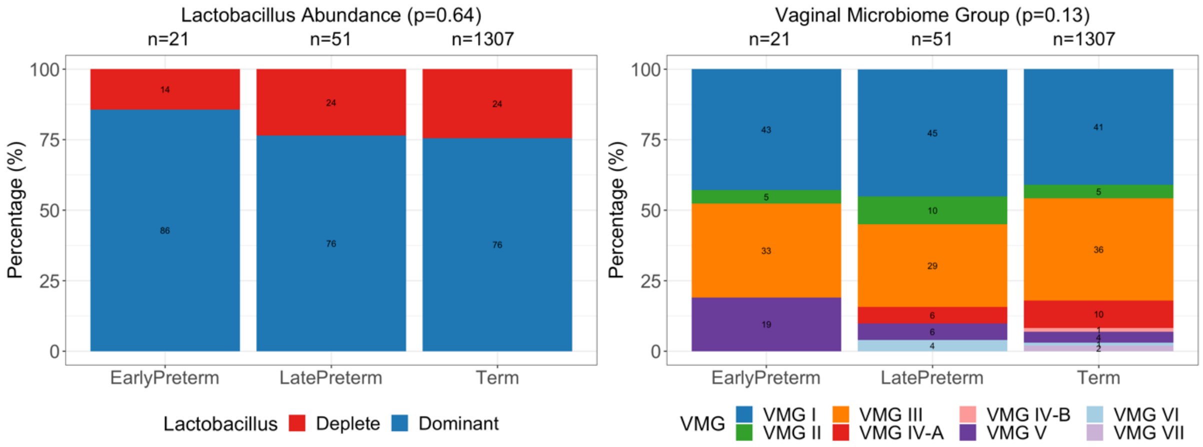

Supplementary Figure 6. Microbiome composition across early preterm, late preterm and term groups based on *Lactobacillus* abundance and vaginal microbiome groups (VMG). Statistical significance based on Fisher's Exact Test.

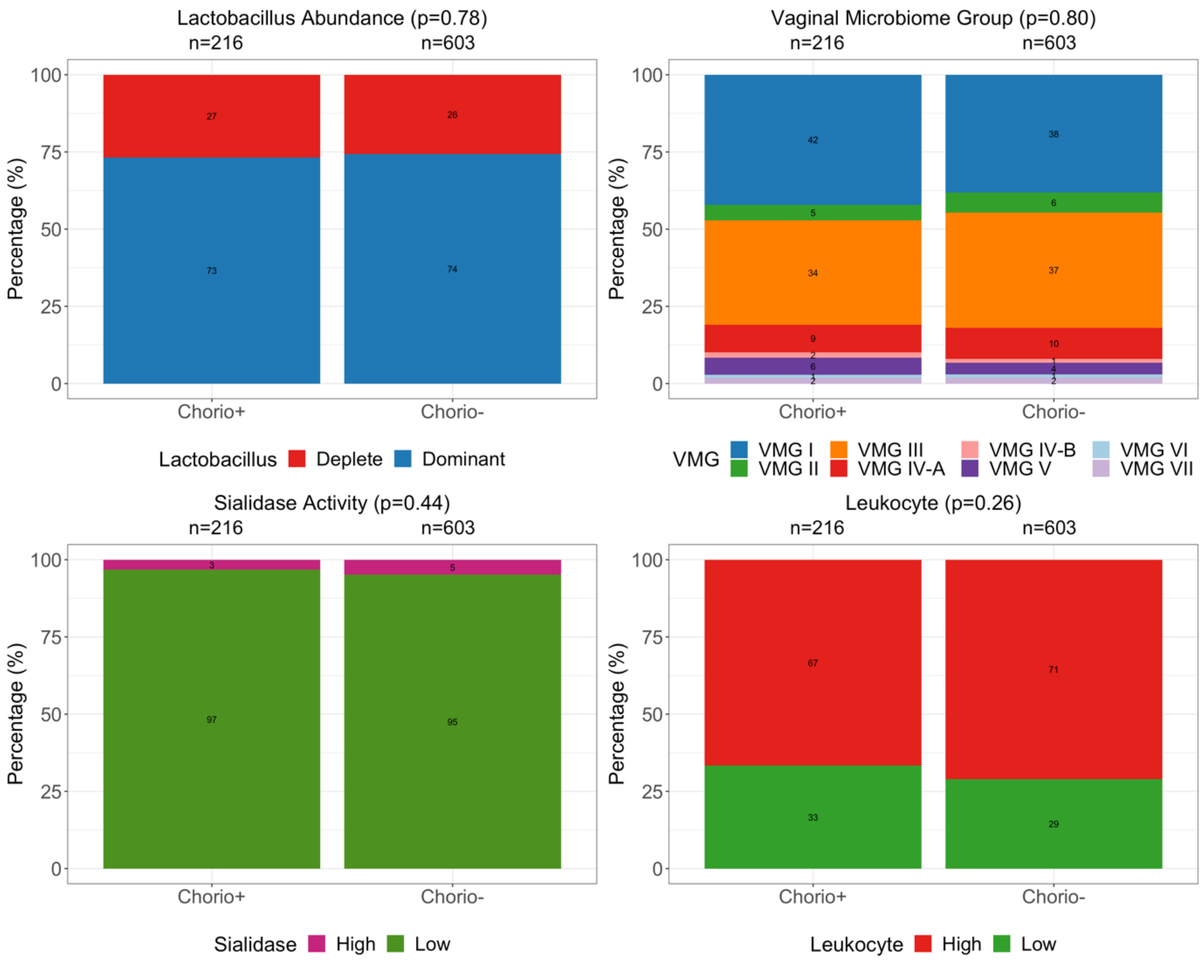

Supplementary Figure 7. Proportions of *Lactobacillus* abundance, VMG, sialidase activity and leukocyte wet mounts based on chorioamnionitis presence (Chorio+) and absence (Chorio-). Statistical significance based on Fisher's Exact Test.

Supplementary Tables

Supplementary Table 1. Relative abundance and Shannon diversity index of dominant species groups.

Dominant Species Group	Relative Abundance*	Shannon Diversity*
*Aerococcus*_spp.	46.12 (45.17 - 47.07)	1.44 (1.34 - 1.53)
*Anaerococcus*_spp.	40.33 (40.33 - 40.33)	1.65 (1.65 - 1.65)
Atopobium_vaginae	64.46 (50.76 - 89.92)	0.87 (0.49 - 1.06)
*Bifidobacterium*_spp.	86.9 (73.01 - 92.94)	0.61 (0.39 - 0.88)
*Gardnerella*_spp.	58.25 (45.40 - 79.30)	1.08 (0.72 - 1.42)
Lactobacillus_crispatus	96.37 (90.35 - 97.97)	0.22 (0.13 - 0.42)
Lactobacillus_delbrueckii	71.08 (56.64 - 75.03)	0.89 (0.78 - 1.08)
Lactobacillus_gasseri	83.73 (63.89 - 93.95)	0.65 (0.33 - 0.88)
Lactobacillus_iners	96.19 (76.41 - 98.14)	0.24 (0.13 - 0.69)
Lactobacillus_jensenii	66.31 (56.38 - 89.41)	0.73 (0.49 - 0.84)
*Lactobacillus*_spp.	75.58 (54.30 - 98.53)	0.72 (0.11 - 0.78)
*Megasphaera*_spp.	35.92 (32.73 - 41.48)	1.57 (1.34 - 1.61)
*Prevotella*_spp.	39.48 (35.63 - 46.25)	1.62 (1.54 - 1.77)
*Shuttleworthia*_spp.	39.60 (33.78 - 50.54)	1.60 (1.28 - 1.65)
Streptococcus_agalactiae	75.90 (60.45 - 85.59)	0.91 (0.62 - 1.20)
Streptococcus_anginosus	69.50 (51.10 - 86.81)	1.04 (0.69 - 1.14)
Streptococcus_luteciae	87.11 (61.32 - 88.65)	0.60 (0.60 - 1.03)
*Streptococcus*_spp.	45.29 (42.93 - 47.65)	1.20 (1.03 - 1.37)
*Veillonella*_spp.	35.87 (35.87 - 35.87)	1.76 (1.76 - 1.76)

*Median (IQR)

Supplementary Table 2. Vaginal microbiome groups (VMG) of women with and without outcome data.

VMG	**Women with available outcome data***	**Women without available outcome data***
I (*L. crispatus* dominated)	567 (41.12%)	491 (38.75%)
II (*L. gasseri* dominated)	70 (5.08%)	67 (5.29%)
III (*L. iners* dominated)	495 (35.90%)	457 (36.07%)
IV-A (BV-associated)	130 (9.43%)	152 (12.00%)
IV-B (Non-BV taxa)	18 (1.31%)	13 (1.03%)
V (*L. jensenii* dominated)	57 (4.13%)	39 (3.08%)
VI (Other *Lactobacillus*)	16 (1.16%)	16 (1.26%)
VII (*Bifidobacterium* dominated)	26 (1.89%)	32 (2.53%)
**Total**	**1379**	**1267**

*number (%)

Supplementary Table 3. Relative abundance of *Lactobacillus*, *Bifidobacterium* and BV-type species in women with high or low sialidase activity.

Genus	High sialidase activity*	Low sialidase activity*	p-value**
Atopobium	1.14 (0.09 - 7.04)	0.09 (0.04 - 0.19)	p<0.01
Bifidobacterium	0.00 (0.00 - 0.03)	0.00 (0.00 - 0.00)	p>0.05
Clostridium	0.00 (0.00 - 0.02)	0.00 (0.00 - 0.00)	p<0.05
Gardnerella	37.99 (9.75 - 61.31)	0.34 (0.20 - 0.73)	p<0.01
Lactobacillus	29.28 (3.19 - 74.68)	98.44 (93.92 - 99.17)	p<0.01
Megasphaera	0.06 (0.00 - 11.62)	0.00 (0.00 - 0.06)	p<0.01
Prevotella	0.98 (0.06 - 10.14)	0.13 (0.05 - 0.33)	p<0.01

*Median (IQR)
**Mann-Whitney Test

Supplementary Table 4. Birth gestation of women based on *Lactobacillus* abundance, sialidase activity and leukocyte wet mount results. No difference in mean gestation at birth was detected between any of the assessed contrasts (Mann-Whitney test).

**Lactobacillus Abundance**		**Sialidase Activity**		**Leukocyte Wet Mounts**		**Birth Gestation (Weeks)***
**Deplete**	**Dominant**	**High**	**Low**	**Leuk+**	**Leuk-**	
x		x		x		39.86<br>(39.29 - 40.50)
	x	x		x		39.14<br>(36.36 - 39.96)
x			x	x		39.43<br>(38.71 - 40.14)
	x		x	x		39.43<br>(38.71 - 40.14)
x		x			x	38.86<br>(38.25 - 39.32)
	x	x			x	39.64<br>(39.61 - 39.68)
x			x		x	39.29<br>(39.00 - 40.14)
	x		x		x	39.29<br>(38.57 - 40.14)

*Median (IQR)

Supplementary Table 5. Logistic regression analysis of women in subcohort (n=819) to determine contribution of *Lactobacillus* abundance, sialidase activity and leukocyte wet mount result to birth outcome.

**Independent variable**	**Logistic regression coefficient**	**Standard error**	**p-value**	**Log-odds**
Lactobacillus Abundance (Deplete = 1, Dominant = 0)	-0.09	0.37	0.81	0.92
Sialidase Activity (High = 1, Low = 0)	0.50	0.66	0.45	1.65
Leukocyte (High = 1, Low = 0)	-0.13	0.33	0.69	0.88
