# Supplementary material for: Large-scale characterisation of the pregnancy vaginal microbiome and sialidase activity in a low-risk Chinese population
Source: NPJ Biofilms Microbiomes. 2021 Dec 20;7:89. doi: 10.1038/s41522-021-00261-0 (PMC8688454; doi:10.1038/s41522-021-00261-0)
Supplement: Supplementary file 3 — Reporting Summary [file 41522_2021_261_MOESM3_ESM.pdf]

nature portfolio

Corresponding author(s): Dr David MacIntyre and Professor Hongwei Zhou

Last updated by author(s): Nov 22, 2021

Reporting Summary

Nature Portfolio wishes to improve the reproducibility of the work that we publish. This form provides structure for consistency and transparency in reporting. For further information on Nature Portfolio policies, see our Editorial Policies and the Editorial Policy Checklist.

Statistics

For all statistical analyses, confirm that the following items are present in the figure legend, table legend, main text, or Methods section.

n/a	Confirmed	
☐	☒	The exact sample size (*n*) for each experimental group/condition, given as a discrete number and unit of measurement
☐	☒	A statement on whether measurements were taken from distinct samples or whether the same sample was measured repeatedly
☐	☒	The statistical test(s) used AND whether they are one- or two-sided<br>*Only common tests should be described solely by name; describe more complex techniques in the Methods section.*
☐	☒	A description of all covariates tested
☐	☒	A description of any assumptions or corrections, such as tests of normality and adjustment for multiple comparisons
☐	☒	A full description of the statistical parameters including central tendency (e.g. means) or other basic estimates (e.g. regression coefficient) AND variation (e.g. standard deviation) or associated estimates of uncertainty (e.g. confidence intervals)
☐	☒	For null hypothesis testing, the test statistic (e.g. *F*, *t*, *r*) with confidence intervals, effect sizes, degrees of freedom and *P* value noted<br>*Give P values as exact values whenever suitable.*
☒	☐	For Bayesian analysis, information on the choice of priors and Markov chain Monte Carlo settings
☒	☐	For hierarchical and complex designs, identification of the appropriate level for tests and full reporting of outcomes
☐	☒	Estimates of effect sizes (e.g. Cohen's *d*, Pearson's *r*), indicating how they were calculated

Our web collection on statistics for biologists contains articles on many of the points above.

Software and code

Policy information about availability of computer code

Data collection	Key analysis code and processed datasets will be available at https://github.com/sherrianne/NgChen_2021_ChinaPregnancyCohort prior to publication.
Data analysis	Raw sequencing data processed using R programming language (v3.4.3) and DADA2 package (v1.6.0). Bioinformatics and statistical analyses performed using R programming language (v4.0.0) and the packages phyloseq (v1.32.0), genefilter (v1.70.0), decontam (v1.9.0), ggplot2 (v3.3.1), corrplot (v0.84), vegan (v2.5-6), edgeR (v3.30.3) and caret (v6.0-86).

For manuscripts utilizing custom algorithms or software that are central to the research but not yet described in published literature, software must be made available to editors and reviewers. We strongly encourage code deposition in a community repository (e.g. GitHub). See the Nature Portfolio guidelines for submitting code & software for further information.

Data

Policy information about availability of data

All manuscripts must include a data availability statement. This statement should provide the following information, where applicable:

- Accession codes, unique identifiers, or web links for publicly available datasets
- A description of any restrictions on data availability
- For clinical datasets or third party data, please ensure that the statement adheres to our policy

Raw data files for the sequence data used in this study are publicly available through the European Nucleotide Archive (https://www.ebi.ac.uk/ena) under accession numbers (PRJNA706523). Relevant patient and clinical data is available as Supplementary Table 1 and at https://github.com/sherrianne/NgChen_2021_ChinaPregnancyCohort.

Field-specific reporting

Please select the one below that is the best fit for your research. If you are not sure, read the appropriate sections before making your selection.

☒ Life sciences ☐ Behavioural & social sciences ☐ Ecological, evolutionary & environmental sciences

For a reference copy of the document with all sections, see nature.com/documents/nr-reporting-summary-flat.pdf

Life sciences study design

All studies must disclose on these points even when the disclosure is negative.

Sample size	Vaginal swab samples were collected from 2796 women. A total of 2689 samples passed library size and vaginal microbiome classification criteria. Only samples collected in the first and second trimester (n=2646) were included in final analyses.
Data exclusions	Sequencing data was excluded based on a pre-established criteria whereby library sizes with <2000 reads were excluded (n=90). Dominant species groups used for microbiome classifications were based on pre-established ≥30% abundance of the dominant species taxon, thus, any samples with <30% abundance of dominant species taxon (n=16) or was classified as an outlier (n=1), were excluded. Of the remaining 2689 samples, samples collected in the third trimester (n=43) were excluded as the vaginal microbiota structure later in pregnancy is different compared to those collected in the mid-trimester.
Replication	Findings were not replicated as only a single vaginal swab sample with 16S rRNA sequencing data was available for each woman included in this study.
Randomization	Samples were randomised prior to sequencing to prevent correlations between run order and outcomes of interest (i.e. preterm and term delivery gestation, sialidase activity, leukocyte wet mount results and chorioamnionitis).
Blinding	Blinding was not relevant as this study did not include different treatment groups.

Reporting for specific materials, systems and methods

We require information from authors about some types of materials, experimental systems and methods used in many studies. Here, indicate whether each material, system or method listed is relevant to your study. If you are not sure if a list item applies to your research, read the appropriate section before selecting a response.

Materials & experimental systems

n/a	Involved in the study	
☒	☐	Antibodies
☒	☐	Eukaryotic cell lines
☒	☐	Palaeontology and archaeology
☒	☐	Animals and other organisms
☐	☒	Human research participants
☒	☐	Clinical data
☒	☐	Dual use research of concern

Methods

n/a	Involved in the study	
☒	☐	ChIP-seq
☒	☐	Flow cytometry
☒	☐	MRI-based neuroimaging

Human research participants

Policy information about studies involving human research participants

Population characteristics	All pregnant women recruited in this study were Chinese, at least 18 years of age, and with or without risk factors for preterm birth. Additional data on population characteristics is available in Supplementary Table 1.
Recruitment	Pregnant Chinese women were recruited at their first prenatal visit from the outpatient clinic of Nanfang Hospital of Southern Medical University, Guangzhou, China. All women were recruited consecutively from January 2015 to December 2018 with no disruptions to recruitment during this period.
Ethics oversight	This prospective study was reviewed and approved ([2013] EC (100)) by the Ethical Committee of Nanfang Hospital, Southern Medical University, Guangzhou, China.

Note that full information on the approval of the study protocol must also be provided in the manuscript.
